# Localisation and Locally Led Development in the Post-consensus Era: Transformation, Stagnation, or Annihilation?

**DOI:** 10.1057/s41287-025-00734-4

**Published:** 2026-01-05

**Authors:** Susan P. Murphy, Maeve McGandy

**Affiliations:** School of Natural Sciences, Geography, Trinity College Dublin, The University of Dublin, Museum Building, Dublin 2, Ireland

**Keywords:** Localisation, Locally led development, International development cooperation, Institutional donor, Critical policy analysis, Localización, Desarrollo liderado localmente, Cooperación internacional para el desarroll, Donante institucional, Análisis crítico de políticas

## Abstract

As the polycrisis of global ecological, political, economic and societal breakdown interacts and unfolds across sites and scales, the need to reform and reorient the international development cooperation regime is urgent. Locally led development (LLD) emerged as a panacea for development ineffectiveness across successive policy paradigms from the Washington Consensus to the Wall Street Consensus. Why such a scalar reorientation is gaining renewed traction, whether this signals a substantive shift towards transformative change in how development is conceptualised and practiced, and how this will influence the development paradigm, remains underexplored. To address these questions, this paper critically analyses the policies and practices of thirty-two institutional donors to assess the extent to which localisation and LLD are recognised and operationalised, enabled and constrained in practice. We offer insights on the current conjuncture and the potentialities within LLD to catalyse disruptive rather than destructive forces and transform development in a post-consensus era.

## Resumen

A medida que la policrisis del colapso ecológico, político, económico y social global interactúa y se despliega en diferentes contextos y escalas, la necesidad de reformar y reorientar el régimen de cooperación internacional para el desarrollo es urgente. El desarrollo liderado localmente (LLD, por sus siglas en inglés) surgió como una panacea frente a la ineficacia del desarrollo a lo largo de sucesivos paradigmas políticos, desde el Consenso de Washington hasta el Consenso de Wall Street. Por qué esta reorientación a nivel de escala está cobrando un renovado impulso, si esto señala un cambio sustantivo hacia una transformación en la manera en que se conceptualiza y practica el desarrollo, y cómo influirá en el paradigma del desarrollo, son cuestiones aún poco exploradas. Para abordar estas preguntas, este artículo analiza críticamente las políticas y prácticas de treinta y dos donantes institucionales para evaluar en qué medida la localización y el LLD son reconocidos y operacionalizados, facilitados y limitados en la práctica. Ofrecemos perspectivas sobre la coyuntura actual y las potencialidades del LLD para catalizar fuerzas disruptivas, en lugar de destructivas, y transformar el desarrollo en una era post-consenso.

## Résumé

Alors que la polycrise résultant des effondrements écologiques, politiques, économiques et sociétaux mondiaux s’entrecroise et se déploie à travers différents sites et échelles, la nécessité de réformer et de réorienter le régime de la coopération internationale au développement devient urgente. Le développement dirigé localement (LLD, par son acronyme en anglais) s’est imposé comme une panacée face à l’inefficacité du développement à travers les paradigmes politiques successifs, du Consensus de Washington au Consensus de Wall Street. Les raisons pour lesquelles une telle réorientation scalaire suscite un regain d’intérêt, la question de savoir si cela traduit un véritable tournant vers un changement transformateur dans la conceptualisation et la mise en œuvre du développement, ainsi que l’influence de cette dynamique sur le paradigme du développement, demeurent peu explorées. Pour répondre à ces interrogations, cet article analyse de manière critique les politiques et pratiques de trente-deux donateurs institutionnels afin d’évaluer dans quelle mesure la localisation et le LLD sont reconnus et opérationnalisés, facilités ou entravés dans la pratique. Nous proposons des éclairages sur la conjoncture actuelle et sur les potentialités du LLD à catalyser des dynamiques disruptives plutôt que destructrices, et à transformer le développement dans une ère post-consensus.

## Introduction and Background

Emerging from the ashes of the Second World War and framed within the international security architecture of the United Nations (UN), the intentional elimination of poverty and the pursuit of prosperity through continuous economic growth via the expansion of a global capitalist economic order has underpinned mainstream international development thought, policy and practice since its inception. Policies and objectives are established internationally and diffused into national contexts. Governance is coordinated through a network of interacting intergovernmental organisations and international financial institutions, supported by governmental and non-governmental organisations operating across geographies and scales (Murphy 2016, 2022).

This hierarchical scalar model has underpinned development cooperation for decades. However, in early 2025, the Trump Regime in the United States of America (US), followed swiftly by other high-income institutional donors including the UK, the Netherlands, and Belgium, signalled a radical dislocation of the sector via drastic reductions in development funding. While the outcome has been a contraction in aid flows, motivations diverge. In the US, reductions reflected an overtly ideological turn toward nationalist retrenchment and disengagement from multilateralism, whereas in Europe cuts were rationalised via technocratic logics of fiscal restraint and efficiency alongside the (re)assertion of domestic, regional, and defence priorities. Despite differing rationales, the combined effect has been a profound erosion of the legitimacy and coherence of the multilateral development system, though the fallout of this rupture is not yet clear. These actions could signal the collapse of the dominant development paradigm, with profound consequences for the poorest and most vulnerable populations, precisely the harms this paradigm was designed to reduce. Alternatively, this moment may serve as a catalyst for the radical transformation of development cooperation governance and practice. Through a critical policy lens, this paper explores the opportunities and threats to development cooperation at this critical inflection point.

Although the idea of ‘development’ is all-encompassing and the concept itself deeply contested, in the context of international development cooperation it broadly refers to a set of processes attached to modernist notions of material progress (Alami et al. 2021). Charting its evolution across time and space, Gillian Hart’s influential distinction between big “D” and little “d” development, drawing upon the theoretical foundations of Polanyi’s double movement and Gramsci’s accounts of hegemony, continues to influence development discourse and debate (Hart 2010). Hart defines “big D” development as ‘the multiple-scale projects of interventions that emerged in the context of decolonization struggles and the Cold War’. Meanwhile, ‘“Little d” development refers to the simultaneous and undergirding ‘expansion of capitalism through geographically uneven but spatially interconnected processes of creation and destruction’ (Hart 2010:119). These interdependent processes produce two distinct forms of praxis in the development sector: one that is predatory and destructive and another that is palliative and caring, providing ‘temporary, uneven and partial redress of the dislocations caused by the creative destruction unleashed by capitalism’ (Mawdsley and Taggart 2022: 5). As observed by Hart and expanded upon by Mawdsley and Taggart (2022), these forms of praxis exist in a dialectical relationship, wherein D/evelopment interventions are deemed necessary to mitigate and alleviate the destruction and suffering caused by the expansion and proliferation of capitalist d/evelopment. In this way, the D/evelopment imperative both sustains and is sustained by d/evelopment.

The concept of ‘locally led development’ (LLD) emerged as a corrective response to the need for a specific rehabilitative form of intervention amid the destruction caused by capitalist expansion and the drive to modernise under the Washington Consensus and its associated structural adjustment programmes of the 1980 s and 1990s. While this paper focuses on the institutionalised, practice-oriented articulation of LLD that emerged within mainstream development discourse during this period, it is important to recognise that ideas of locally grounded or endogenous development have a much longer and more heterogenous intellectual lineage, and that prominent institutional interpretations did not emerge in isolation. Earlier and diverse trajectories of critical development thought, from Latin American Marxist and dependency traditions (e.g. Mariátegui 1988), to postcolonial and decolonial geographies and Southern critiques of development (Escobar 1995, 2012; Slater 1993), had long articulated demands for a reconfiguration of the hierarchical and spatial logics of development itself, challenging the North-South binaries through which mainstream development has historically been theorised and practiced. These diverse theoretical and political movements provided much of the normative and analytical groundwork later appropriated within institutionalised LLD agendas, even as their redistributive and structural critiques were diluted through their translation into policy frameworks (Gómez 2021; Roepstorff 2020). This paper acknowledges these broader genealogies while centring its analysis on LLD’s contemporary articulation within dominant policy frameworks.

Early iterations of the concept featured prominently in the World Bank’s endeavours to enhance the overall effectiveness of development practice (World Bank, 1989), characterised by four domains of action: social capital strengthening, participation in development processes, decentralisation, and civil society building (Mohan and Stokke 2008). A central focus within the Development Effectiveness debates of the 2000 s (OECD 2023), LLD was positioned as a means of ensuring development was locally owned and driven, embedded within social cooperation at all scales. The term ‘localisation’ emerged later, featuring prominently in the context of debates around the in/effectiveness of humanitarian action, pointing to the urgent need to recognise and realise the capacities of local and national actors as first responders in humanitarian emergencies (Barakat and Milton 2020). Although the governing principles, practices, and institutional architecture of development and humanitarian action are distinct, financial flows and their overarching policy architectures are intertwined and interdependent, articulated through development cooperation policies at national and international scales.

The UN’s Agenda 2030 and the Sustainable Development Goals (SDGs) have, until recent interventions by the Trump Regime in the United States, constituted the global policy architecture governing contemporary international development cooperation. Building on earlier frameworks and agendas such as the Human Development Index (HDI) and the Millennium Development Goals (MDGs), Agenda 2030 extends a longer trajectory through which development thinking has moved beyond a singular focus on economic growth toward a multidimensional understanding of wellbeing, inclusion, and sustainability. These successive agendas redefined the logics and parameters through which development has been practiced and measured. Outlining distinct global goals, the SDG framework marks a departure from the previously entrenched spatial and discursive distinction between countries of the so-called Global North and South, and from dichotomous framings of ‘developed’ and ‘developing’ regions, states, and locales. Countries are conceived as being positioned along a broadly linear development trajectory, one which purportedly integrates and balances environmental and ecological considerations with economic and social transformations (Horner 2020). Despite this globalised framing, the enduring Global North-South development model has persisted (Doane 2024), influenced by Western capitalist development histories and norms and governed through professionalised networks of experts across sites and scales with accountability for outcomes focused more heavily on donors in the Global North rather than affected communities in target countries in the so-called Global South (Murphy et al. 2019, #shiftthepower 2024). Such activity is largely funded through official development assistance (ODA), the financial lifeblood of the sector and long recognised as a core instrument of statecraft used to build and sustain affinities among populations geographically distant, but strategically important for the economic growth and national security of donor states. In 2023, ODA flows reached approximately $223.7 billion USD (OECD 2024). This figure declined in 2024 and is expected to fall sharply again in 2025.

As a process shaped by intersecting economic, political and spatial forces, critical development scholars explain how international d/Development cooperation rests upon asymmetrical power structures embedded within contingent Global North-South relations (Sakue-Collins 2021), reinforced through dynamic geographies of production and consumption (Escobar 2012, Ziai 2007, Artner and Yin 2023). Persistent power asymmetries have spurred recurrent resistance and calls for change emanating within and beyond institutional and state frameworks, questioning and challenging not only to what is done but also how, by whom, to whom, and for whom. A recent OECD report captures the interrogative disassembling impetus behind such calls, noting how ‘anti-racist movements call to upend colonial legacies and a renewed push for locally led development have triggered reflections on how the aid system is structured and operates’ (2023: 18). Despite recognition of the need for change, little research examines how institutional donors support, finance, and facilitate LLD as a mechanism for transforming development practice (Banks 2021; OECD 2024). This paper addresses this lacuna by critically examining how institutional donors conceptualise and operationalise LLD and localisation in policy through their commitments, funding modalities, and ODA allocations.

The following section begins with a brief historical overview of LLD and localisation within development. Examining conceptual and operational shifts across distinct eras of development policy, we trace their evolution, identify factors influencing their endurance as donor priorities, and question why both concepts remain largely unactioned. Section three outlines the methodological framework used to analyse 102 policy documents issued by 32 institutional donors. Section four outlines and discusses the key themes that emerged throughout our analysis. We conclude with some critical observations on the current conjuncture. Departing from constructions of LLD as a standalone technical instrument, we attend critically to the assemblage dynamics evident within framings of LLD and localisation. These point to a range of unstable, dynamic, contingent process and relations wherein opportunities for transformation are embedded within and revealed through ruptures to contemporary d/Development as a necessarily local, place-based range of practices.

## The History and Evolution of LLD and Localisation

Commencing with the movement towards participatory approaches in the 1970 s (Cornwall and Scoones (eds) 2022), and emerging in mainstream development policy in the late 1980 s with the collapse of the Soviet Union and the end of the first Cold War, LLD has continued to feature heavily within development discourse as a necessary component of effective and efficient cooperation (Walsh 2025). Emphasising the agency and ownership of local actors in responding to economic development and determining d/Developmental outcomes, early articulations of LLD framed it as a means of engaging affected populations and communities as participants in the transformation of their lands, landscapes and livelihoods amidst the expansion of an increasingly globalised, market-driven model of economic growth.

Over time, different approaches to development practice emerged which attempted to deepen participation and local engagement, including ‘doing development differently’, ‘adaptive development’, and ‘thinking and working politically’ (Craney and Hudson 2020). Development, however, has always been an unavoidably local practice, unfolding within distinct historical, political, and ecological contexts, even as it is influenced by global processes. Ensuring local support for and ownership of interventions therefore seems an obvious necessity to securing their acceptability and sustainability over time. Although contested and deeply multiplicitous, LLD is broadly understood as a process: a way of doing or approaching development, rather than a distinct goal or endpoint. The OECD define LLD as co-operation that recognises and supports local actor agency across four key aspects of development interventions: framing, design, delivery (including control over resources), and accountability (OCED DAC 2023: 4). Recent reports call for more extensive analysis of institutional donor practices to better understand the structural and contextual factors shaping current approaches to LLD across geographies (OECD, 2024).

While LLD has featured prominently for almost four decades, the term ‘localisation’ emerged more recently, predominantly mobilised within the context of humanitarian action, where it generally refers to the aim of ‘strengthening local ownership through equitable partnerships and redistribution of resources and decision making’ (Novovic 2021: 1). Explicitly framed within the Grand Bargain, a formal policy declaration emerging from the 2016 World Humanitarian Summit, institutional donors are called upon to provide quality funding and greater support to the leadership, delivery and capacity of local responders (The Grand Bargain (Official website) | IASC).

According to Vij (OECD 2023), contemporary articulations of LLD and localisation draw on various instrumental, normative, and emancipatory rationales. Proponents claim localisation enhances efficiency, assuming that locally sourced labour, resources, and materials cost less than their international counterparts (Koch and Rooden 2024; Robillard et al. 2021; Barakat and Milton 2020). Others argue local actors have a deeper understanding of the specificity of local needs and a better comprehension of culturally appropriate responses. Some commentators argue that such instrumentalist perspectives obscure the explicitly political nature of localisation, rooted in calls for the radical decolonisation of the sector (Currion 2020; Tawake et al. 2021; Peace 2022). Others argue that such approaches grossly misrepresent ‘the local’ as a disembodied, ahistorical, homogenous entity divorced from the diverse, multiplicitous realities of populations affected by place-based d/Development (Barakat and Milton 2020; Roepstroff 2020).

From a normative perspective, LLD and localisation are increasingly framed as corrective approaches to development, indicating a direction from which interventions ought to be designed and implemented to centre the autonomy and agency of affected populations (Slim 2021). Embedded within such framings is a recognition that LLD and localisation are fluid, relational processes, influenced by distributed agencies and pluralities of power across cooperating actors and an unquantifiable range of endogenous and exogenous factors. Both concepts discursively signal a more favourable approach to development practice, rather than explicitly determining what ought to be done, who ought to be involved, and how.

Key policy moments have punctuated LLD’s evolution as a development priority. First articulated in the World Bank’s 1989 report, LLD discourses resurfaced during the 2000 s Aid Effectiveness debates (OECD, 2024), culminating in the Paris Declaration, which centred ownership and encourages partner countries to ‘take the lead in their own development processes’ (OECD 2005: 3). Ownership and inclusive partnerships re-emerged again in 2011, featured prominently within the Busan Partnership and the Global Partnership for Effective Development Cooperation (GPEDC), launched at the Fourth High-Level Forum on Aid Effectiveness jointly hosted by UNDP and OECD.

Within the humanitarian sector, the principle of consent and the recognition of local actors as first responders in humanitarian crises is embedded within the 1991 UN General Assembly (UNGA) Resolution 46/182, which centres consent of affected populations and underscores the importance of demand-led action specified by affected states. Such sentiments are encapsulated in the UN Inter Agency Standing Committee’s position, which notes that localisation ‘enables the meaningful engagement and leadership of local and national actors in the humanitarian response, enhancing capacity exchange and increasing direct funding’ (Inter-Agency Standing Committee, n.d.). Over time, a shift in discourse away from consent and country-led response towards more expansive calls to ‘make humanitarian responses more efficient, effective, adequate, inclusive and emancipatory’ (Roepstorff 2020: 285) are evident across the literature.

Interrogating the logics underpinning LLD’s emergence and persistence as a mechanism for enhancing development effectiveness reveals two key justifications: legitimacy and leverage, alongside classical economic arguments in favour of assumed efficiency based on lower local labour costs. As the creative-destructive forces of globalised neoliberal capitalism expandedand unfolded throughout the 1990 s and 2000 s, new mechanisms were required to mitigate the harm borne by populations in ‘developing’ countries while building consent and commitment to the emerging dominant institutionalised social order (Fraser 2013, 2023; Hart 2010). Legitimacy for this new political economic order was constructed through efforts to socialise, naturalise, and internalise capitalist norms, beliefs, and behaviours that valorised individualism, self-interest and competition over the collective wellbeing. Following the devastation of structural adjustment, it became strategically essential to furnish capitalist expansion with a more humane face that could promote its ‘benefits’ and soften its harshest effects. This period marked the beginning of large flows of financing to and through non-governmental and civil society organisations to reach those most marginalised through these processes via big “D” development interventions (Mohan and Stokke 2008; Gaynor 2022; Sakue-Collins 2021).

As the post-Washington consensus period extended through the 2000 s, further leverage was required to socially embed and creatively expand market logics and capitalist values to reach places and populations ‘left behind’. Development interventions experimented with blending ‘participatory approaches’ and ‘economic empowerment’ to bring the most marginalised members of communities into formal productive labour and financial markets through capacity building, training, empowerment programmes, and micro-financing, all of which naturalised and further legitimised the dominant globalised neoliberal capitalist hegemony (Carroll and Jarvis 2015; Bebbington et al., 2008).

More recent shifts in development discourse towards deep marketisation (Carroll and Jarvis 2015) and financialisaton that Gabor (2021) refers to as the ‘Wall Street Consensus’ mark a distinct turn from the state to the private sector. Given the estimated $4 trillion USD needed to achieve the SDGs (United Nations Interagency Taskforce of Financing for Development, 2024), institutional donors are increasingly exploring opportunities for public-private partnerships to design and deliver development interventions by leveraging private financing and encouraging private investment, with donors assuming a lead role in de-risking such investments (Alami and Dixon 2024). As this shift unfolds, d/Development processes continue to maintain and sustain the need for D/development interventions (Mawdsley and Taggart 2022; Sakue-Collins 2021). Within this context, LLD and localisation have reemerged as necessary means of ensure the effectiveness of interventions.

## Methods and Materials

Much of the research on localisation and LLD has focused on the work of international non-governmental organisations (INGOs) and civil society organisations (CSOs) (Currion 2020; Peace 2022) given their central role in designing and implementing big “D” development interventions, and their dynamism in simultaneously engaging with affected populations and local and national actors across contexts. Despite their role in agenda-setting, there has been little systematic analysis of institutional donor positions on this topic (Banks et al. 2020; OECD 2023). This research is thus guided by a relatively simple question: to what extent do institutional donors recognise and realise LLD and localisation in and through their development policy architecture?

To answer this question, we examine individual donor positions, analysing how commitments around LLD and localisation are interpreted, communicated, and intended to be implemented – by whom, with whom, and for whom. Given the normative and deeply ideological language of development cooperation, critical analysis of donor statements is essential to reveal their underlying logics and assumptions, and theoretical implications. Drawing on elements of Fairclough’s critical discourse analysis (2013) and Jessop’s cultural political economy (CPE) (2010), we use these frameworks as interpretive lenses rather than as methodological templates. Fairclough’s emphasis on discourse as a site where ideology and power are enacted guided our reading of how donors construct and legitimise their commitments to LLD, while Jessop’s CPE highlights how such constructions intersect with broader political-economic rationalities. We trace the performative dimensions of donor policy texts by identifying themes and conceptualisations that characterise distinct, often competing accounts of LLD and localisation. We also examine practical indicators, such as references to international agreements, shifts in funding patterns, and the framing of financial flows. Our analysis focuses less on linguistic or semiotic intricacies and more on the broader discursive construction of reformist ideas and promises, alongside their omissions and silences, to expose the gaps, contradictions, and foreclosures shaping institutional articulations of LLD and localisation.

Following Thomas et al. (2007), the policy review proceeded in two stages to select policies for inclusion and develop a typology of institutional donors’ positions on LLD and localisation. Firstly, publicly available policy and strategy documents on development cooperation and humanitarian aid – issued by government ministries or national development agencies up to early 2024 – were analysed. This stage covered 64 documents from 32 institutional donors. Initial selection and review highlighted the need to expand the search to include policies and positions on civil society, as INGOs and CSOs are identified by most institutional donors as key partners in the operationalising localisation and LLD. A second stage therefore examined civil society-related policies, which contained more extensive context and guidance for enhancing local participation in development practice. In total, 104 documents and position papers reviewed, of which 56 contained specific information relevant for analysis.

Following the initial descriptive analysis, Gaynor’s *4 W* Framework was applied to examine the power dynamics embedded in each institutional donor’s policy architecture, focusing on the ‘where’, ‘ways’, ‘what’, and ‘who’ of LLD and localisation (2022: 51). Grounded in a poststructuralist understanding of power, institutions, and agency, the framework offered a lens for analysing how authority, status, and socio-spatial relations shape the language and meaning of LLD and localisation across donors. This approach facilitated an examination of both visible and subtle expressions of power, ranging from explicit formal commitments and financial flows to the positioning of decision-making processes. It guided questions such as: at what stage are local and national actors engaged, and for what purpose? In what ways are affected populations represented, considered, or consulted? Who participates in framing, implementation, and evaluation, and for whom? Finally, from an ideological perspective, we interrogate the hegemonic ideals, norms, and assumptions that these texts articulate and promote.

## Constraints and Limitations

Several key challenges emerged during the review, driven by rapid and ongoing shifts in the geopolitical landscape that carry significant implications for national political contexts and are influencing donor positions on aid and financial flows. Conflict in Ukraine and Gaza, coups across the Sahel, the collapse of Sudan, and unrest in the DRC, South Sudan, Kenya, Bangladesh, Turkey, and elsewhere are driving the making, unmaking and remaking of approaches to development cooperation, with renewed emphasis on risk, values, and interests. More broadly, the legitimacy and functionality of the international system have been challenged and called into question in light of the paralysis and deliberate undermining of key multilateral institutions, such as the UN, in responding to these crises.

Major political transitions are also reshaping the international development cooperation landscape. In early 2025, a major shift in US policy under the Trump administration triggered widespread disruption across the sector, prompting other donors to reassess aid commitments and priorities. This rupture accelerated longer-term funding contractions, deepened uncertainty within the system, and exacerbated humanitarian pressures in regions already under severe strain. Though the longer-term implications of these shifts remain unclear, and most formal policy frameworks are yet to be revised, this review provides a timely baseline against which future policy and practice changes can be measured and assessed.

## Results and Discussion

The following section critically examines how localisation and LLD are conceptualised and operationalised within contemporary institutional donor policies, interrogating the extent to which such policies engage in any meaningful reorientation of power and resources towards more locally derived forms of development practice. The first subsection engages with performative indicators, analysing *how* localisation is interpreted and framed by different institutional donors. We identify a series of competing narratives embedded within and across individual texts. At a more granular level, we scrutinise which donors reference their signatory status in relation to international commitments and statements on localisation and LLD. This facilitates an examination of how various donors embed their positionality on localisation and LLD within their development policy frameworks, either explicitly or implicitly. By exploring the key emergent themes, we identify points of convergence and divergence in how donors situate themselves within and beyond localisation and LLD discourses. The second subsection analyses practical indicators, assessing how donors operationalise their commitments. We examine the identification of key partners, intended delivery mechanisms, financial commitments, and actual financial flows to evaluate how and to what extent policy commitments are enacted in practice. The findings discussed offer critical insights into the motivations and logics underpinning current donor positions on localisation and LLD. They illuminate the underlying priorities, discourses and strategic considerations shaping various approaches, highlighting key factors enabling and inhibiting the operationalisation of such ideas within development policy and practice.

## Conceptualising LLD and Localisation - Examining Performative Indicators

### Explicit Positioning: Acknowledging Signatory Status

Two major international agreements on localisation feature prominently across the corpus: the Grand Bargain and the Donor Statement on Supporting Locally led Development (USAID, 2022). The Grand Bargain, launched at the 2016 World Humanitarian Summit, commits signatories to strengthen locally led responses and increase support for local and national actors as first and front line responders in emergencies. Its key target, under work package 2 on localisation, is to channel at least 25% to such actors; at the time of writing, 24 of 32 OECD DAC donors are signatories (a full list of signatories can be found here). The Donor Statement, introduced at the 2022 Global Partnership on Effective Development Cooperation summit in Geneva and co-led by USAID and the Norwegian development agency NORAD, calls for shifting funding and decision making power to local actors and organisations through reliable, direct, multi-annual, and flexible financing. At the time of writing, 21 out of 32 institutional donors have endorsed this agreement.

Although similar in intent, these agreements set different expectations for donors and engage distinct institutions, organisations, and interests across humanitarian and development spheres. The Grand Bargain (2016) aims to strengthen cooperation among key stakeholders, amplify local and national voices, and increase funding to local actors in humanitarian contexts. The Donor Statement (USAID 2022b) calls for more direct financing of national and local actors but offers little clarity on how transnational partnerships might be structured or governed. Two broad approaches thus emerge in the scalar, spatial, and regulatory practice of development: direct and indirect localisation.

As shown in Table 1, different sets of institutional donors align with different agreements, indicating distinct geopolitical orientations and reflecting diversity in their positions. Six institutional donors have not signed either agreement. However, signatory status alone does not signal a coherent or consistent position on LLD, nor does its absence infer anti-LLD sentiment. All donors articulate identifiable positions on LLD, though they differ significantly in how they interpret and frame such ideals, shaped by their own self-defined regulations, development priorities, and interventionary logics.

**Table 1:** Status of institutional donor signatories to relevant agreements

International statement	Institutional donor signatories
Grand Bargain (24/32)	Australia, Belgium, Canada, Czech Republic, Denmark, **EU**, Estonia, Finland, France, **Germany**, Ireland, **Italy**, Japan, South Korea, **Luxembourg**, the Netherlands, **New Zealand**, Norway, Slovenia, Spain, Sweden, Switzerland, United Kingdom, United States of America
Donor statement on LLD (21/32)	Australia, Belgium, Canada, Czech Republic, Denmark, Estonia, Finland, France, **Iceland**, Ireland, Japan, South Korea, **Lithuania**,** the Netherlands**, Norway, Slovenia, Spain, Sweden, Switzerland, United Kingdom, United States of America
Neither (6/32)	**Austria**,** Greece**,** Hungary**,** Portugal**,** Poland**,** the Slovak Republic**

### Interpreting Localisation

Our analysis found that only six out of 32 institutional donors explicitly define localisation within their development cooperation policies (Australia, Belgium, the EU, Italy, the Netherlands, and the US), though interpretations vary considerably. All reviewed policies were published or updated after the 2016 Grand Bargain, and most followed the 2022 Donor Statement. Situating our analysis within this temporal frame highlights how localisation has been unevenly incorporated into donor policy architectures, with varying levels of responsiveness to these sectoral commitments. In the Italian context, localisation implies a business-as-usual approach through ‘continuing to support local actors, which play the lead role in emergency response’ (Italy, 2021: 41), while the EU and Australia are more pointedly concerned with capacity strengthening of local and national actors (DG ECHO 2023; Australia 2021). The US and the Netherlands indicate a focus on revising internal operations to enhance the position of local actors (USAID 2023a; the Netherlands 2022), with both citing internal reform of their governmental and non-governmental agencies is a necessary prerequisite. The Dutch Ministry of Foreign Affairs, for instance, links localisation to organisational diversity and notes that ‘development organisations’ advertisements must not stigmatise, supporting rather than undermining local self-reliance’ (2022:39).

USAID emerged as the most ambitious institutional donor in framing localisation, defining it as ‘the set of internal reforms, actions, and behaviour changes that the Agency is undertaking to ensure our work puts local actors in the lead, strengthens local systems, and is responsive to local communities […]’ (USAID 2023a). They specify four priority areas to advance localisation in practice: adapting policies and embedding programmes more effectively in local socio-political and economic contexts, shifting power to local actors, challenging a larger proportion of funds through to local and national actors, and advocating globally to influence other agencies to follow suit. Their emphasis on internal reform and the direct targeting of local and national organisations as partners represents the most significant effort to operationalise localisation to date (Table 2).

**Table 2:** Key themes emerging within and across institutional donor positions

Theme	Institutional donor policy/Position
**(Th1)** Recognising Local Autonomy, Agency & Ownership **(19/32)**	Australia, 2021; Austria, 2022; Belgium, n.d.; Czech Republic, 2017; Denmark, 2021; DG ECHO, 2023; Finland, 2023; France, 2018; Greece, 2022; Iceland, 2022; New Zealand 2020; Norway, 2018; Poland, n.d.; Slovakia, 2019; Slovenia, 2018; Spain, 2023; Sweden, 2018, 2013; UK, 2022; US 2923b, 2022b
**(Th2)** Prioritising Localised, Demand-Oriented Practice **(18/32)**	Australia, 2023; Austria, 2022; Canada 2017; DG ECHO, 2023; Estonia, 2020; Finland, 2023; Hungary 2020, 2014; Italy, 2021; Japan, 2011; South Korea, 2022; Lithuania, 2021a; Luxembourg, n.d.; The Netherlands, 2022; New Zealand 2022a; Poland, n.d.; Slovenia, n.d.; Sweden 2013, 2018; Switzerland, 2021
**(Th3)** Ensuring Participation **(15/32)**	Denmark, 2021; DG ECHO, 2023; Finland, 2023; Germany, 2023; Hungary, 2020; Ireland 2022, 2019; Italy, 2021; Luxembourg, n.d.; the Netherlands, 2022; New Zealand 2022a; Norway, 2018; Portugal, 2023; Slovakia, 2019; Slovenia n.d.; UK, 2022
**(Th4)** Enhancing Mechanisms for Localising Development/strengthening local ownership (**13/32)**	Australia, 2023; Canada, n.d.; DG ECHO, 2023; Estonia, 2020; Germany, 2023; Italy, 2021; Luxembourg, 2022; the Netherlands, 2022; New Zealand 2022a; Norway, 2018; Sweden, 2024; UK, 2022; US, 2023a, b, 2022b.
**(Th5)** Alignment with Recipient Country Development Priorities **(7/32)**	Czech Republic, 2017; Estonia, 2020; Iceland, 2022; Lithuania 2021b; Slovenia, n.d.; Spain, 2023; Sweden 2017;
**(Th6)** Foregrounding Indigenous Epistemologies **(8/32)**	Australia, 2023; DG ECHO, 2023; Finland, 2017; Japan 2022; Luxembourg, 2022; the Netherlands, 2022; New Zealand 2022b; Sweden, 2013
**(Th7)** Foregrounding Inclusivity **(5/32)**	Belgium, n.d; Finland 2023, 2021; Ireland 2022, 2019; Luxembourg, 2022; New Zealand 2022a;

Analysis of 56 development cooperation policy documents identified seven dominant themes, revealing contradictions and discursive tensions across donor profiles. The most frequent theme (Th1) concerns recognition of local actors’ autonomy and the importance of local ownership. Interestingly, only ten donors connected this recognition to supporting and enabling local leadership (Th4), and thirteen donors explicitly prioritised local participation (Th3). A second major theme (Th2) highlights that interventions should be demand-driven and responsive to local needs, though only seven donors explicitly plan to align their practices with recipient states’ national development plans (Th5). The remaining themes expose deficiencies in ethical implementation practices. Just five donors noted explicit commitments to inclusivity as a condition of LLD (Th7), and eight acknowledge the need to respect and integrate indigenous knowledges within their practices (Th8). These findings underscore deep and persistent discursive tensions in how LLD imagined and operationalised.

As with signatory status, donor positions on operationalising LLD and localisation fall into two categories. The first approach, initially led by USAID, invokes a need for deep institutional and practical shifts, recognising the potential of LLD as a mechanism for driving structural transformation within the aid system. This approach entails disassembling traditional ways of doing development cooperation and humanitarian action and reassembling more emancipatory forms of practice. Attention is directed toward reorienting internal processes to enable direct engagement with local entities, translation of materials into local languages, and supporting capacity assessments of local entities to assess the scope for direct financing. Donors focus on adapting projects and programmes to better fit practice contexts and seek out new sets of local and national actors, agencies, and voices throughout the development practice cycle. A distinguishing feature of this approach is the decreasing prominence of international organisations. Overall, there is an identifiable impulse to redistribute power between practitioners, but not between donor and beneficiary.

The second approach is less oriented towards structural change, instead focusing on strengthening funding to local and national actors indirectly through existing partnerships with INGOs and IGOs, pre-screened and pre-certified to manage grants and reporting. This model involves no regulatory reform to enable direct financing of local actors; its main operational focus is capacity development. Around 20 donors reference capacity building as a means to ‘empower’ local actors and increase their participation in development cooperation. Other, less common mechanisms include expanding or deepening partnerships (Austria 2023, EU ECHO 2023, Germany 2023, Ireland, 2019, Luxembourg n.d., the Netherlands 2022) strengthening dialogue (France 2021, Italy 2021, Spain 2023), supporting locally based organisations (Denmark 2021), promoting flexible and innovative approaches (Australian Government 2023, EU ECHO 2023, Ireland 2019), enhancing transparency (Australia 2023, Austria 2022, Lithuania 2021a), and increasing support for decentralisation (EU ECHO 2023, Spain 2023). Spain (2023) and the EU (2023) commit to simplifying bureaucratic procedures, while Switzerland (2021) seeks to challenge negative stereotypes of local actors’ capacities. Overall, this approach positions LLD as a mechanism to stabilise and maintain the existing aid system by enhancing local skills and ensuring local actors’ involvement across project design, implementation, and evaluation (DG ECHO 2023).

Though distinct, both approaches remain entangled within and dependent upon the existing ODA superstructure, with little evidence of critical reflection or structural transformation of the mechanisms that reproduce uneven development and power asymmetries. Rooted in an outdated unipolar global order, the system’s core institutions and processes remain largely untouched by LLD efforts. As the polycrises of global economic, ecological, political, and societal breakdown intensify across sites and scales, their harshest effects continue to fall on those with the least capacity to adapt, deepening multidimensional poverty and further excluding marginalised voices from decisions that shape the most intimate aspects of their lives (UNDP 2024). These converging crises demand more adaptive, equitable, and accountable systems of development practice.

None of the approaches analysed adequately conceptualise or mobilise an account of the ‘local’ that sufficiently captures its heterogeneity. Donor policy frameworks, transposed into national and local development planning processes, remain insufficient for framing interventions as necessarily local, place-based matters. Across all policies, the ‘local’ is presented as vague and ambiguous, represented as an abstracted, homogenous category stripped of its socio-political and contextual complexity. A substantial body of scholarship has long interrogated ‘who’ can be considered local and how this is influenced by race, colonisation, and migration (Tawake et al. 2021), as well as organisational form (Roepstorff 2020). Such insights are absent from donor framings, even as our analysis reveals dynamic, contested, and heterogenous local landscapes and the unavoidably political tensions that operate within and across them.

Within donor discourse, the ‘local’ remains under-theorised and, to an extent, romanticised. From a socio-spatial perspective, it constitutes a distinctly heterogeneous, complex, relational sphere imbued with diverse social structures and power dynamics, and attributed meaning through embedded cultures, norms, and practices. The richness, inherent opportunities, and multiple geographic imaginaries entailed within the ‘local’ (MacGinty 2015) are flattened through the language of LLD as a technical process of resource redistribution. Combined with existing power asymmetries, LLD shapes how decisions are made and whose knowledge counts. These arenas are alive with competition, conflicting interests, and alternative development propositions and possibilities (Mohan and Stokke 2008). However, within the established aid architecture, the superstructure of development cooperation appears cemented. Here, local agency operates inside donor-defined boundaries, with capacity development positioned as the main route to participation. As Goodwin and Ager (2021) show, requiring local organisations to ‘build capacity’ to compete for funding reinforces the idea that local knowledge systems are less valuable and compels assimilation into dominant systems to ensure participation.

Our analysis shows that institutional donors largely fail to acknowledge the asymmetrical power relations underpinning development cooperation policies and offer few alternative mechanisms to balance interests or redistribute decision-making power. Across the corpus, structural barriers remain embedded in two ways. Firstly, through continued support for established systems of managing development. Secondly, through resistance to revising the regulations governing the flow and management of aid. On the first point, a tacit consensus emerges around maintaining professionalised development practices such as result-based management, which reinforce upward accountability to donors rather than enabling mechanisms that might shift accountability towards affected populations (Murphy et al. 2019 on Ireland’s results-based agenda and Valters and Whitty’s (2017) on international applications).

On the second point, the funding regulations of several donors, such as the European Commission and many of its member states, including Ireland, explicitly exclude direct financing of local organisations, requiring funds to flow through established partners and networks, including pre-approved INGOs, IOs, and via pool-funding mechanisms. These structural barriers constrain transformative LLD practices and largely foreclose the potential to redistribute power and decision-making to those at the centre of development and humanitarian interventions. The discursive ambiguity surrounding LLD enables powerful actors to interpret and engage with it selectively to advance their own interests. Rather than realising its transformative promise, the commitment to emancipation functions as a mechanism for reproducing dominance and preserving the hierarchical superstructure that has long underpinned development cooperation.

## Operationalising Localisation Responsibilities: Analysing Practical Indicators

### Financial Commitments

The Grand Bargain and LLD commitments specify that 25% of funds should be re/distributed to and through local and national actors. How donors plan to do differs substantially, as outlined in Table 3.

**Table 3:** Funding and financing locally led development

Actions:	Institutional donors:
Increasing and improving financing	Australia, EU, France, Germany, Luxembourg, The Netherlands, New Zealand, Norway, Slovenia, Switzerland, US.
Multi-year funding	Australian Government, Canada, Germany, EU, Ireland
UN Pooled funding/funding to centralised calls	EU, Canada, Luxembourg, Norway, Ireland
Direct funding to donor based strategic partners – support on-granting/partnership arrangement with L/Nos	EU; Austria; Australia; Belgium, Denmark, Ireland, the Netherlands, New Zealand (requirement that negotiated partnerships include a 50% transfer of overhead funds/management costs to local partner), Norway, Slovenia
Joint partnerships between Donor country and Local/National organisation/Consortia	Belgium, the Netherlands
Funding through Embassy networks	Austria, Hungary, Ireland, France, Finland, the Netherlands
Direct support to local/national civil society organisations	Denmark, France, Germany, (local self-government institutions) US, Sweden, Iceland (selected partners and programme participants)
Include localisation markers (25% target) in monitoring and reports	US, EU (through strategic partners), France

**Fig. 1:**
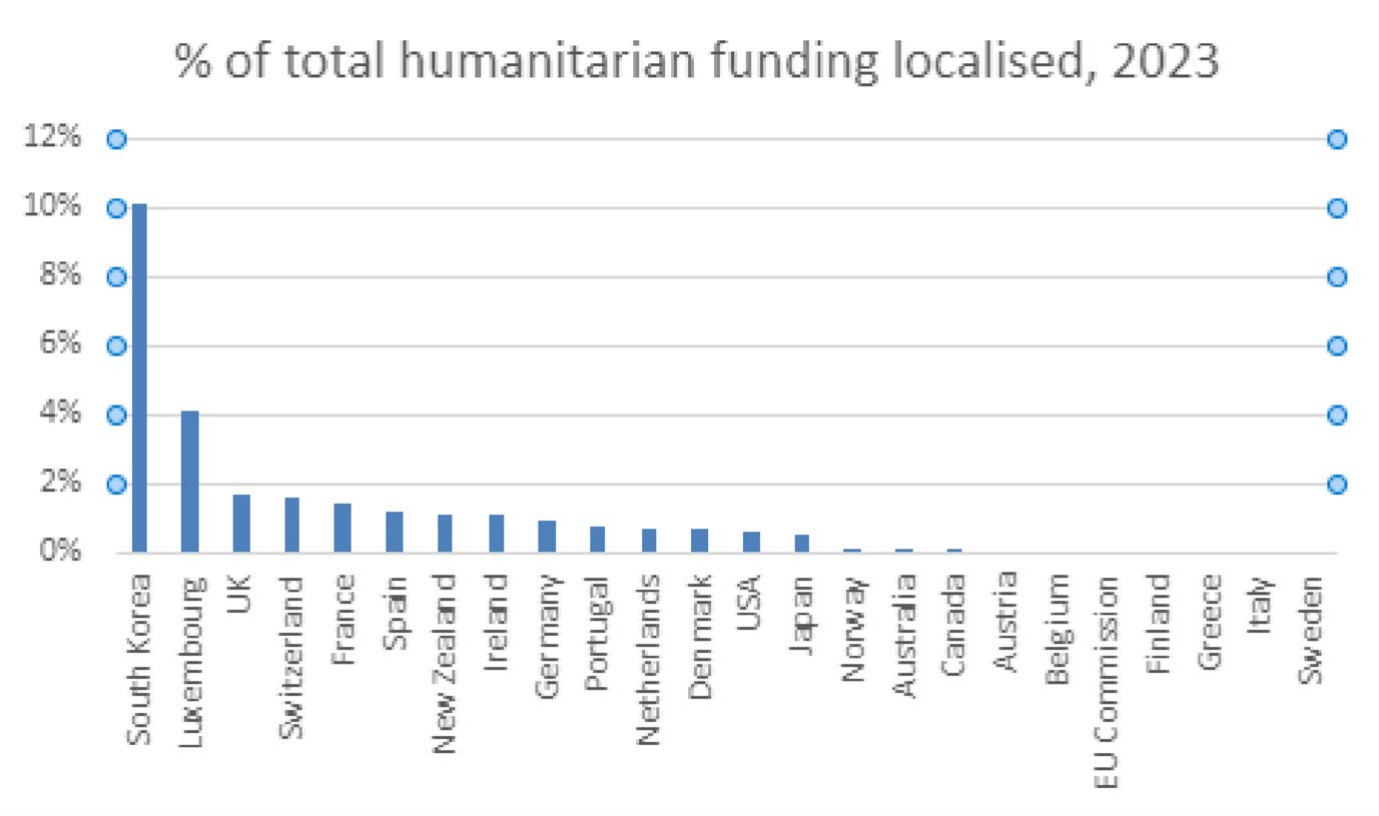
Institutional donor breakdown of humanitarian aid flows (OCHA FTS 2024)

**Fig. 2:**
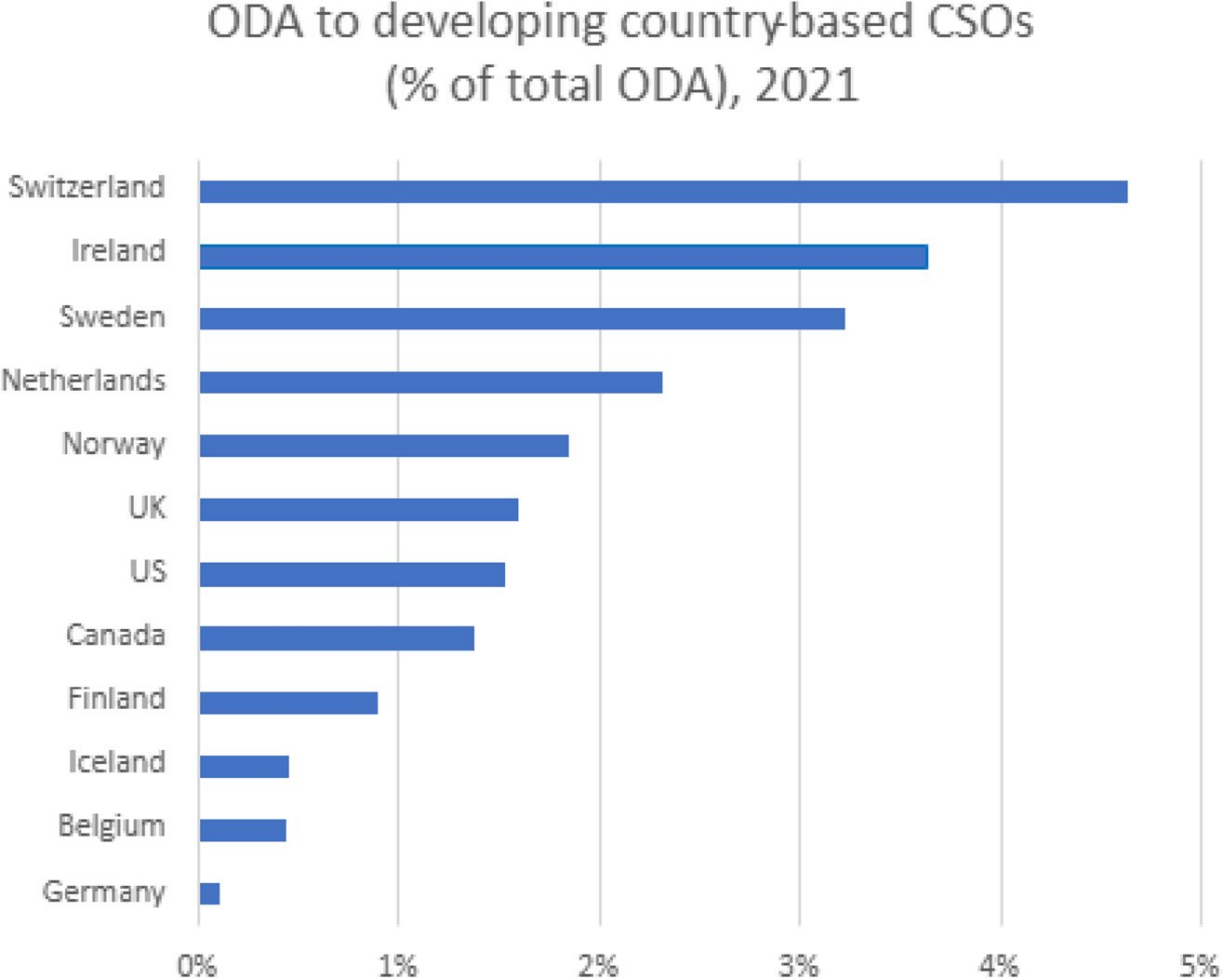
Total ODA to developing country based CSOs and national/local actors by institutional donor (source OECD Statistics)

Wading through the range of performative and elaborative descriptions of the myriad of ways in which enhanced and more direct funding can be achieved, data provided in the *Global Humanitarian Assistance Report* (Development Initiatives 2023) indicate an overall decline in funds flowing to local and national actors since the emergence of the Grand Bargain (2016) falling from a high of 3% in 2018 to its lowest point of 1.2% in 2023. Data from the Financial Tracking Service of the Office for the Coordination of Humanitarian Affairs (2024) provides a further breakdown by institutional donor. The figures reveal a stark inconsistency between donors’ discursive commitments and their actual funding patterns, where smaller donors such as South Korea and Luxembourg lead in terms of localised humanitarian funding (Fig. 1), while Switzerland and Ireland rank highest in development funding disbursements (Fig. 2).

Given that development interventions are typically planned over several years, with intended impacts spanning decades, and transnational relationships established and sustained over time, the persistently low volume of funding reaching local and national actors – directly and indirectly – reflects a broader among dominant actors and interest groups to embrace structural change. Capacity building efforts have been ongoing, yet local and national actors continue to act as implementers of externally designed projects rather than as architects of development pathways for their own local and national populations.

## Conclusions

In many ways, the latest policy articulations of LLD and localisation represent continuity with historical and institutional antecedents, wherein the ‘local’ generally remains homogenised and radically underappreciated. For most institutional donors, resource redistribution primarily appears to occur within and between actors and organisations already dominant within the sector, with little indication as to how this could or should change. A lack of incentives for a radical revision of internal processes further limits opportunities for a broader, more expansive engagement with local and national actors. Within our analysis, the US emerged as an outlier in this case, explicitly examining revisions to their internal processes and structures to better facilitate direct financial flows to local and national actors. However, all has since changed for USAID. Other donors, including the UK, France, Belgium, the EU, and the Netherlands are actively reducing ODA financing at a time in which the international order is dissolving, and needs continue to proliferate. The sector appears locked in a vicious cycle, as crises at once amplify needs and directly erode the capacities of the institutions charged with meeting them. Further, as change unfolds across the sector, the risks of unintended consequences accompanying these shifts remain under-examined, and the potential for harm in such scalar reorganisation remains high (Murphy 2016). Beyond this, calls for ‘equitable’ and ‘joint’ partnership approaches fail to engage with the political and normative dimensions shaping institutional donor conceptions of LLD. As resources decline, competition among local, national, and international organisations is likely to intensify, risking a further weakening of transnational solidarity and cooperation.

For most institutional donors, despite the subtle promises within their conceptualisations of LLD, our analysis reveals cleat instances of co-option and depoliticisation in how such ideas are operationalised. Rather than engaging with LLD’s normative implications or critically assessing the extent to which current practices enhance or dilute the autonomy of local and national actors, most policies adopt a technocratic framing. This approach risks obscuring donor intent and reinforcing the asymmetrical power structures embedded within the contemporary development cooperation regime. Indeed, the latest indications emerging from the OECD peer learning exercise on LLD (2023) point to the persistence of disembodied, ahistorical, depoliticised assumptions informing institutional donor practices and policies, albeit wrapped in a veneer of enlightened national interest and opportunities for mutual benefit (OECD 2023). These tendencies toward depoliticisation and technocratic drift reflect not only institutional logics but also deeper transformations in the architecture of development governance. Financialisaton has redefined development cooperation around risk management, efficiency, and market-oriented practice, rather than emphasising commitments to redistribution or solidarity. As institutions retreat and private actors assume greater influence, donors’ capacities to reimagine mechanisms of cooperation have declined. This failure is not merely institutional but structural, an effect of the wider expansion and contraction cycles of global capitalism. The implications therefore extend beyond the technical scope of LLD to question the very configuration of development governance itself.

Such shifts aside, the reasons for the re-emergence of this turn to the local do not appear to have radically changed. Needs for legitimacy and leverage remain high. As d/Development processes continue to expand, D/development interventions remain necessary to address some of the most severe effects and reinforce the legitimacy of capitalist expansion and the institutional social order that this entails (Fraser 2023). D/development financing remains a lever of soft power and influence for Western states, albeit at rapidly declining rates. As geopolitical shifts continue to challenge Western hegemony, institutional donors have explicitly sought approaches to development cooperation that allow them to re-position themselves as “partners of choice” in response to the rise of South-South cooperation and China’s expanding role in international development cooperation (Alami et al. 2021; UK 2023, EU 2021). This era of development cooperation is marked by a declining and fracturing consensus (Niblett 2024). As conversations around the era of a new cold war amplify, it can be argued that we have entered a post-consensus era. What this implies for the post WWII development cooperation architecture remain unclear and uncertain. Despite evidence of resistance to change among dominant actors in the sector, the post-consensus era also presents opportunities to reimagine relations and approaches to development practice, with LLD offering one potential entry point. As the US withdraw, and traditional institutional donors decrease funding, needs remain and continue to accelerate. This is likely to result in a turn to and greater reliance on local actors, organisations, and solutions.

Our analysis indicates that while renewed interest in localisation and LLD shows some promise for shifting the scalar configuration of intentional, interventionist big “D” Development, the scope and reach of immanent capitalist little “d” development remain largely untouched. International development cooperation remains in its traditional form, anchored in particular spaces and places (Gómez 2021). The potential for more meaningful LLD is yet unrealised but continues to rest in the possibilities entailed in the multiplicity and diversity of the ‘local’. Actions undertaken in development and humanitarian practice are always unavoidably local. They are embedded within local histories, politics, societies, and ecologies. Conceptions of development that seek to flatten and reduce this dynamism, diversity, and difference risk remaining trapped within the entrenched big “D”/little ”d” dialectic. To ignore such practical reality is to risk condemning practices of aid and development cooperation to incoherence and irrelevance.

### List of Policy Materials and Resources

**Table:** 

Institutional donor	Year of publication	Document link or title
Australia	2021	https://www.dfat.gov.au/development/who-we-work-with/ngos/ancp/australian-ngo-cooperation-program
	2023	Australia’s International Development Policy | Australian Government Department of Foreign Affairs and Trade
Austria	2022	https://www.bmeia.gv.at/fileadmin/user_upload/Zentrale/Aussenpolitik/Entwicklungszusammenarbeit/Three-Year_Programme_on_Austrian_Development_Policy_2022-24.pdf.
Belgium	n.d.	https://www.enabel.be/who-we-are/our-strategy/.
Canada	2017	https://www.international.gc.ca/world-monde/assets/pdfs/iap2-eng.pdf?_ga=2.146638141.882574840.1725738978-2068843130.1712526199.1725738978.1712526199.
	n.d.	https://www.international.gc.ca/world-monde/issues_development-enjeux_developpement/priorities-priorites/civil_policy-politique_civile.aspx?lang=eng.
Czech Republic	2017	https://mzv.gov.cz/file/2710363/CZ_Development_Cooperation_Strategy_2018_2030.pdf.
Denmark	2021	https://reliefweb.int/report/world/world-we-share-denmark-s-strategy-development-cooperation.
DG ECHO	2023	https://interagencystandingcommittee.org/sites/default/files/migrated/2023-03/EU%20DG%20ECHO%20guidance%20note%20-%20Promoting%20equitable%20partnerships%20with%20local%20responders%20in%20humanitarian%20settings.pdf.
Estonia	2020	1.1 Välisministeerium-arengukava-juuni_EN_valmis.indd
Finland	2017	https://um.fi/documents/35732/48132/the_guidelines_for_civil_society_in_development_policy_2017.
	2021	https://julkaisut.valtioneuvosto.fi/bitstream/handle/10024/163334/UM_2021_05.pdf?sequence=1&isAllowed=y.
	2023	https://julkaisut.valtioneuvosto.fi/bitstream/handle/10024/165144/UM_2023_13.pdf?sequence=1.
France	2023	CICID 2023: France’s new strategy for international development cooperation
	2018	releve_de_conclusions_du_comite_interministeriel_de_cooperation_internationale_et_du_developpement_-_08.02.2018_cle4ea6e2-2.pdf
Germany	2023a	https://www.bmz.de/en/issues/feminist-development-policy.
	2023b	https://www.auswaertiges-amt.de/blob/2585076/4d2d295dad8fb1c41c6271d2c1a41d75/ffp-leitlinien-data.pdf.
Greece	2023	https://www.mfa.gr/images/docs/Strategic%20Plan%202023-2026_EN.pdf.
Hungary	2014	https://nefe.kormany.hu/download/3/93/c0000/International%20Development%20Cooperation%20and%20Humanitarian%20Aid%20Strategy%20of%20Hungary-v%C3%A9gleges.pdf.
	2020	https://nefe.kormany.hu/download/7/3f/92000/NEFE2025_summary_en.pdf.
Iceland	2018/2019	https://www.government.is/library/01-Ministries/Ministry-for-Foreign-Affairs/Int.-Dev.-Coop/Publications/Parliamentary%20Resolution%20on%20Iceland%e2%80%99s%20policy%20for%20international%20development%20cooperation.pdf.
	2022	https://www.government.is/library/01-Ministries/Ministry-for-Foreign-Affairs/Int.-Dev.-Coop/Publications/EN%20-%20Civil%20Society%20Organization%20Cooperation%20Strategy.pdf.
Ireland	2008	https://www.irishaid.ie/media/irishaid/allwebsitemedia/20newsandpublications/publicationpdfsenglish/civil-society-policy.pdf.
	2019	https://www.ireland.ie/en/irish-aid/news-and-publications/publications/publication-index/a-better-world-irelands-policy-for-international-development/.
	2022	https://www.irishaid.ie/media/irishaid/whatwedo/whoweworkwith/internationalorganizations/Multilateral-Operational-Framework-FINAL-for-website.pdf.
Italy	2021	https://www.esteri.it/wp-content/uploads/2022/07/Programming-and-policy-planning-document-2021-2023.pdf.
Japan	2011	https://www.mofa.go.jp/policy/emergency/pdfs/Outline_hap.pdf.
	2022	https://www.jica.go.jp/Resource/english/publications/reports/annual/2022/fh2q4d000001doiv-att/2022_all.pdf.
Korea (Republic of)	2022	‘Office for International Development Cooperation Republic of Korea (2022) Strategic Plan for Official Development Assistance under the Yoon Administration, pp. 1–21’ (no date).
Lithuania	2021a	https://ltaid.urm.lt/en/doclib/wzul6ssdrk9j49dd6gpbx1rggnft8nu8.
	2021b	https://www.urm.lt/storage/main/public/uploads/2024/02/development-cooperation-strategy-for-africa-20222025-.pdf.
Luxembourg	2022	https://cooperation.gouvernement.lu/dam-assets/publications/strat%C3%A9gies/action-humanitaire/luxembourgs-humanitarian-action-strategy.pdf.
	n.d.	https://cooperation.gouvernement.lu/content/dam/gouv_cooperation/publications/strat%c3%a9gies/strategie-generale/Strat%c3%a9gie-MAEE-EN.pdf.
the Netherlands	2022a	https://www.government.nl/documents/policy-notes/2022/10/10/policy-document-for-foreign-trade-and-development-cooperation-do-what-we-do-best.
	2022b	https://www.government.nl/documents/policy-notes/2019/11/28/policy-framework-strengthening-civil-society.
New Zealand	2022	https://www.mfat.govt.nz/assets/Aid/4YPs-2021-24/Humanitarian-4-year-plan.pdf.
	n.d.	https://www.mfat.govt.nz/assets/Aid-Prog-docs/Policy/New-Zealands-International-Development-Principles.pdf.
Norway	2018	"https://www.norad.no/postmottak@norad.no.
Poland	n.d.	https://www.gov.pl/web/polishaid/the-multiannual-programme-for-development-cooperation-for-2021-2030-solidarity-for-development.
Portugal	2023	https://diariodarepublica.pt/dr/detalhe/resolucao-conselho-ministros/121-2022-204502329.
Slovak Republic	2019	https://slovakaid.sk/wp-content/uploads/2021/01/strednodoba_strategia_rozvojovej_spoluprace_eng_2019-2023_644_stran_final.pdf.
Slovenia	2018	https://www.gov.si/assets/ministrstva/MZZ/Dokumenti/multilaterala/razvojno-sodelovanje/ZMRSHP_EN-final.pdf.
	n.d.	https://www.gov.si/assets/ministrstva/MZZ/Dokumenti/multilaterala/razvojno-sodelovanje/Development-Cooperation-and-Humanitarian-Aid-Strategy-of-the-Republic-of-Slovenia.pdf.
Spain	2023	https://www.boe.es/eli/es/l/2023/02/20/1/con.
Sweden	2013	https://www.regeringen.se/contentassets/6eef64a9a36e48ff9a95e4d6ad97ce84/aid-policy-framework.
	2017	https://www.government.se/contentassets/3291aeacc48c495898d5bd59702d9e32/guidelines-for-strategies-in-swedish-development-cooperation-and-humanitarian-assistance.pdf.
	2024	Sida’s operational strategy 2024–2026
Switzerland	2021	https://www.eda.admin.ch/deza/en/home/sdc/publications.html/content/publikationen/en/deza/diverse-publikationen/broschuere-iza-2021-24.
UK	2022	https://www.gov.uk/government/publications/uk-governments-strategy-for-international-development/the-uk-governments-strategy-for-international-development.
	2023	International development in a contested world: ending extreme poverty and tackling climate change – A White Paper on International Development
US	2022	https://www.state.gov/wp-content/uploads/2022/03/Final-State-USAID-FY-2022-2026-Joint-Strategic-Plan_29MAR2022.pdf.
USAID	2022a	‘USAID (2022a) Donor Statement on Supporting Locally Led Development, retrieved at Donor Statement on Supporting Locally Led Development \textbar Basic Page \textbar U.S. Agency for International Development (usaid.gov)’
	2022b	Localisation at USAID: The Vision and Approach, pp. 1–5’ (no date). Available at: https://www.usaid.gov/sites/default/files/2022-12/USAIDs_Localization_Vision-508.pdf.
	2023a	‘USAID (2023a) Localisation, Catalyzing and supporting local change, retrieved at Localization \textbar What We Do \textbar U.S. Agency for International Development (usaid.gov)’ (no date).
	2023b	Moving toward a model of locally led development, FY 2022 Localisation Progress Report, pp. 1–23’ (no date). Available at: https://www.usaid.gov/sites/default/files/2023-06/FY%202022%20Localization%20Progress%20Report-June-12-23_vFINAL_1.pdf.
